# Microbiota and Derived Parameters in Fecal Samples of Infants with Non-IgE Cow’s Milk Protein Allergy under a Restricted Diet

**DOI:** 10.3390/nu10101481

**Published:** 2018-10-11

**Authors:** María Díaz, Lucía Guadamuro, Irene Espinosa-Martos, Leonardo Mancabelli, Santiago Jiménez, Cristina Molinos-Norniella, David Pérez-Solis, Christian Milani, Juan Miguel Rodríguez, Marco Ventura, Carlos Bousoño, Miguel Gueimonde, Abelardo Margolles, Juan José Díaz, Susana Delgado

**Affiliations:** 1 Department of Microbiology and Biochemistry of Dairy Products, Instituto de Productos Lácteos de Asturias (IPLA)-Consejo Superior de Investigaciones Científicas (CSIC), 33300 Villaviciosa, Spain; Maria.Diaz@quadram.ac.uk (M.D.); luciagg@ipla.csic.es (L.G.); mgueimonde@ipla.csic.es (M.G.); amargolles@ipla.csic.es (A.M.); 2 Department of Nutrition and Food Science, Universidad Complutense de Madrid (UCM), 28040 Madrid, Spain; irene.espinosa@probisearch.com (I.E.-M.); jmrodrig@ucm.es (J.M.R.); 3 Department of Chemistry, Life Sciences and Environmental Sustainability, University of Parma, 43121 Parma, Italy; leonardo.mancabelli@genprobio.com (L.M.); christian.milani@unipr.it (C.M.); marco.ventura@unipr.it (M.V.); 4 Pediatric Gastroenterology and Nutrition Section, Hospital Universitario Central de Asturias (HUCA), 33011 Oviedo, Spain; principevegeta@hotmail.com (S.J.); ringerbou@yahoo.es (C.B.); 5 Pediatrics, Hospital Universitario de Cabueñes, 33394 Gijón, Spain; cristinamolinos@gmail.com; 6 Pediatrics, Hospital Universitario San Agustín, 33401 Avilés, Spain; doctorin@gmail.com

**Keywords:** fecal microbiota, protein hydrolyzed formulas, cow’s milk protein, tolerance acquisition, non-IgE mediated allergy

## Abstract

Cow’s milk protein allergy (CMPA) is the most common food allergy in infancy. Non-IgE mediated (NIM) forms are little studied and the responsible mechanisms of tolerance acquisition remain obscure. Our aim was to study the intestinal microbiota and related parameters in the fecal samples of infants with NIM-CMPA, to establish potential links between type of formula substitutes, microbiota, and desensitization. Seventeen infants between one and two years old, diagnosed with NIM-CMPA, were recruited. They were all on an exclusion diet for six months, consuming different therapeutic protein hydrolysates. After this period, stool samples were obtained and tolerance development was evaluated by oral challenges. A control group of 10 age-matched healthy infants on an unrestricted diet were included in the study. Microbiota composition, short-chain fatty acids, calprotectin, and transforming growth factor (TGF)-β_1_ levels were determined in fecal samples from both groups. Infants with NIM-CMPA that consumed vegetable protein-based formulas presented microbiota colonization patterns different from those fed with an extensively hydrolyzed formula. Differences in microbiota composition and fecal parameters between NIM-CMPA and healthy infants were observed. Non-allergic infants showed a significantly higher proportion of Bacteroides compared to infants with NIM-CMPA. The type of protein hydrolysate was found to determine gut microbiota colonization and influence food allergy resolution in NIM-CMPA cases.

## 1. Introduction

Cow’s milk protein (CMP) is the main cause of food allergy in the first year of life. Based on the involvement of IgE antibodies, CMP allergy (CMPA) is classified as classic IgE-mediated, non-IgE mediated (NIM), and mixed pathophysiology [1]. Typically, clinical presentation is delayed, and digestive symptoms are more frequent, with NIM-CMPA than with IgE-mediated CMPA [2]. In both types, treatment involves the avoidance of cow’s milk formula and the use of a therapeutic formula as a substitute. Clinical guidelines recommend the use of an extensively hydrolyzed formula (EHF) as the first choice, while amino acid-based formulas are reserved for those cases not responding to an EHF [3]. Soy formulas represent an option in infants older than six months [4], and rice formulas have been safely used during the last few years [5]. Nowadays, a wide number of articles highlight the importance of the correct establishment/development of gut microbiota in early life and its impact on allergic diseases, including food allergies [6,7]. Although some studies have related intestinal microbiota and allergy resolution in IgE-mediated CMPA [8,9], research on gut microbiota exclusively in NIM-CMPA has been hampered by the difficulty of diagnosis [10]. However, differences in microbial composition between infants with IgE-mediated and non-IgE mediated food allergies have been reported [11]. The present study was designed to evaluate intestinal microbiota and fecal associated parameters in infants with NIM-CMPA, under a milk elimination diet, compared to healthy infants, with an unrestricted conventional diet, with the aim of establishing potential links among microbiota, main feeding sources, and tolerance acquisition.

## 2. Materials and Methods

### 2.1. Subjects

Infants 12 to 24 months old diagnosed with NIM-CMPA (*n* = 17) were prospectively recruited for study participation at three different regional hospitals in Asturias (Northern Spain): *Hospital Universitario Central de Asturias, Hospital Universitario de Cabueñes, and Hospital Universitario San Agustín.* All participants had symptoms suggestive of CMPA, a negative skin prick test, values lower than 0.35 kU/L cow’s milk-specific IgE determined in their blood, and a clear positive standardized oral challenge (SOC), performed under medical supervision following the European Society for Pediatric Gastroenterology, Hepatology, and Nutrition (ESPGHAN) guidelines. Infants that were exclusively breastfed at the diagnosis, and those that had used antibiotics or had symptoms of an infectious disease in the four weeks prior to the stool sample collection, were excluded from the study.

The study was approved by the Regional Ethics Committee for Clinical Research of Principality of Asturias (Ref. number 105/15, approved on 22 June 2015). Personal data of the children that provided stool samples conformed to the ethical guidelines outlined in the Declaration of Helsinki and its amendments. Individual signed informed consents were obtained from all the families participating in the study.

### 2.2. Study Design

This was a prospective cohort study. A detailed medical history, including type of feeding and formula used, were recorded by the clinicians. All infants were on a cow’s-milk-free diet for at least six months before a new SOC was performed. Following the elimination diet period, and before the new SOC, stool samples were collected. A control group of 10 age-matched healthy infants (range 12–24 months old), with a normal diet consuming CMP, were included in the study and provided stool samples. Feces from all the participants were collected by their parents in sterile containers and immediately frozen at −20 °C. All samples were thawed on ice once they had been delivered to the laboratory and processed accordingly for different analyses.

### 2.3. Intestinal Microbial Community Analysis

Extraction of DNA from feces was based on the method of Zoetendal et al. [12] using the QIAamp DNA Stool mini kit (Qiagen, Hilden, Germany), with some modifications as previously described [13]. Partial 16S rRNA gene sequences were amplified from the DNA of the samples according to previous reports [14]. Samples were submitted to 2 × 250 bp paired-end sequencing by an Illumina MiSeq System (Illumina, San Diego, CA, USA). All Illumina quality-approved, trimmed, and filtered sequences were processed using a custom script based on the QIIME software suite [15]. Sequences were classified to the lowest possible taxonomic rank considered (i.e., genus level), using QIIME and the SILVA database as reference. Weighted UniFrac was employed to assess the similarity of the microbial communities between infants. The raw sequences data were deposited in the Sequence Read Archive (SRA) of the NCBI (https://www.ncbi.nlm.nih.gov/sra) under accession numbers SRR6884553 to SRR6884580.

### 2.4. Analysis of Short-Chain Fatty Acids (SCFAs)

A chromatographic system, composed of 6890N gas chromatography (GC) apparatus (Agilent Technologies, Santa Clara, CA, USA) connected to a flame ionization detector (FID), was used. All samples were analyzed in duplicate and SCFAs were quantified as previously described [16].

### 2.5. Calprotectin Assays

Calprotectin levels were determined using the commercially available enzyme-linked immunosorbent assay (ELISA) kit CALPROLAB^TM^ (Calpro, Lysaker, Norway) according to the manufacturer’s instructions.

### 2.6. Transforming Growth Factor-β_1_ (TGF-β_1_) Determination

The concentration of TGF-β_1_ in the feces was determined by using a Bio-Plex 200 system instrument and the Bio-Plex Pro^TM^ TGF-β Assay (both from Bio-Rad, Hercules, CA, USA). Analysis of samples was carried out as previously described [17].

### 2.7. Statistical Analysis

Statistical analysis of microbiological sequences was performed with the Metastats statistical method [18]. Multiple hypothesis tests were adjusted using a false discovery rate (FDR) correction of 0.25. Multivariable statistical analysis was performed by principal coordinates analysis (PCoA) and the plot was visualized in the EMPeror Visualization Program [19]. Differences in the microbial distribution between infants were sought by analysis of molecular variance (AMOVA). Biochemical fecal data were analyzed using IBM SPSS 23 statistic software. Normality was checked by the Shapiro–Wilk test. As the variables were not normally distributed, medians and interquartile ranges (Q1 and Q3) were calculated, and comparisons were performed, by using the non-parametric Mann–Whitney test. The level of significance was set at *p* values of <0.05. Finally, the Spearman correlation method was conducted to elucidate the relationship between the variables of the study.

## 3. Results

Seventeen infants (nine male and eight female) were recruited into the NIM-CMPA group and 10 infants into the control group. No statistically significant differences were found with respect to sex and age between the two groups: median of 17 (13–23) months in the NIM-CMPA group vs. 18 (14.3–24) months in the control group. Infants in the NIM-CMPA group were fed with EHFs (12 patients, 70.6%), soy protein-based formulas (two patients, 11.8%), and hydrolyzed rice formulas (three patients, 17.6%). All of them developed tolerance to CMP by the end of the study with the exception of the three infants fed a rice formula.

### 3.1. Microbiota Analysis in NIM-CMPA in Relation to Tolerance and Diet

**Figure 1:**
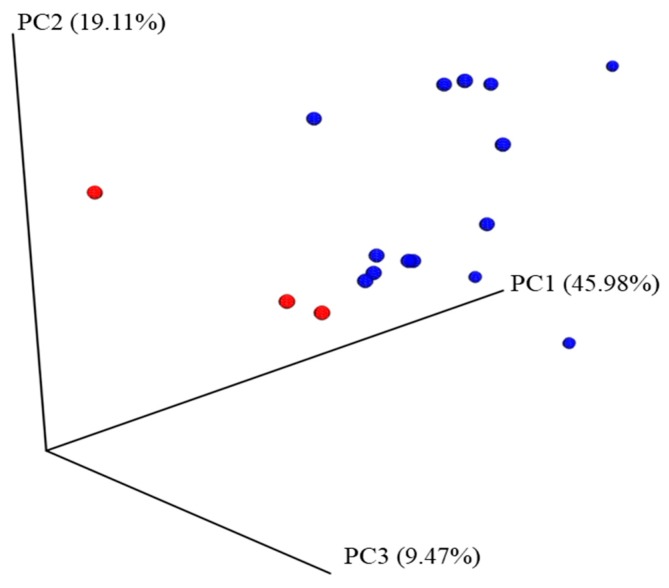
Principal coordinates analysis (PCoA) plot. For its construction, the weighted Unifrac method was used to compare the bacterial communities among samples from non-tolerant (*n* = 3) and tolerant (*n* = 14) infants with non-IgE mediated cow’s milk protein allergy (NIM-CMPA), based on their phylogenetic relationship. Percentages shown in the axes represent the explained variance. Blue circles illustrate samples from tolerant infants whereas red circles illustrate those that maintain active hypersensitivity after the standardized oral challenge (SOC). Analysis of molecular variance (AMOVA) was used to assess the statistical significance of the spatial separation between both groups (*p* = 0.02).

The three infants who did not develop tolerance to CMP, after the exclusion diet for six months, presented a clear distinct microbiota colonization pattern characterized by a low abundancy of sequences of Actinobacteria, in particular the genus *Bifidobacteria* (Table 1). Distinctively, the infant that reported the quickest and severest gastrointestinal (GI) symptoms after the SOC presented a clear dysbiotic pattern, with a marked presence of sequences belonging to the phylas Verrucomicrobia and Proteobacteria (infant code 12; Figure S1). The PCoA plot showed that the samples from the three non-tolerant infants did not clearly cluster and were separated from the infants who acquired desensitization to CMP (Figure 1). However, when AMOVA was used to assess the statistical significance of the spatial separation, significant differences in the microbial clustering between tolerant and non-tolerant infants (the latter ones consuming rice formula) were revealed (*p* value = 0.02).

Other members of the Actinobacteria phylum, in particular the family *Coriobacteriaceae*, was significantly diminished in the infants with NIM-CMPA that consumed vegetable protein-based formulas (*n* = 5; mean of 0.64% of total assigned reads; range 0.02–2.38%), both rice and soy, compared to those infants fed with an EHF (mean of 4.45%; range 0.08–13.44%).

### 3.2. Microbiota Analysis Comparison between the NIM-CMPA and Control Groups

**Figure 2:**
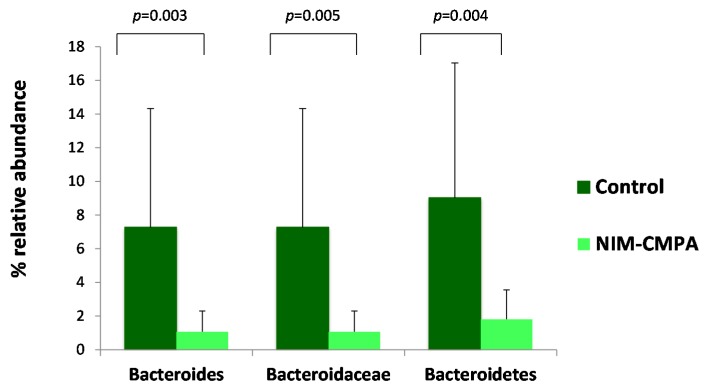
Differences in relative abundances (%) of sequences belonging to the phylum Bacteriodetes, the family *Bacteroidaceae*, and the genus *Bacteroides* in fecal samples of infants with non-IgE mediated cow’s milk protein allergy (NIM-CMPA) (*n* = 17) and non-allergic control infants (*n* = 10). Comparisons were corrected with a false discovery rate (FDR) of 0.25.

Differences in the composition of the gut microbial communities between the infants with NIM-CMPA and the infants in the control group were observed. The non-allergic group, following an unrestricted diet, showed a significantly higher proportion of sequences of the phylum Bacteroidetes, the family *Bacteroidaceae*, and the genus *Bacteroides* compared to the NIM-CMPA group (Figure 2). In contrast, some members of the Clostridiales order, such as the *Eubacterium fissicatena* group (of the family *Lachnospiraceae*), were significantly higher in allergic infants, which also included the aforementioned *Coriobacteriaceae* family (mean of 3.33% of assigned reads, ranging from 0.02% to 13.44%, in the NIM-CMPA group versus 0.86% of assigned reads, ranging from 0.01% to 4.12%, in the control group).

### 3.3. Fecal Excreted SCFAs

Regarding the quantification of the SCFAs in the feces, there were no statistically significant differences in the acetic, butyric, and propionic levels between the NIM-CMPA and control groups (Table 2). For butyric acid, levels were higher in the infants with NIM-CMPA; however, the difference was borderline and not statistically significant (*p* = 0.06). There was a positive correlation (Spearman correlation coefficient *r* = 0.48, *p* = 0.01) between butyric acid levels and fecal *Coriobacteriaceae*, both of which were higher in the allergic group (see Figure S2).

Conversely, the branched chain fatty acids (BCFAs), isobutyric and isovaleric acids, presented significantly higher levels in the infants with NIM-CMPA than in the control group (*p* = 0.03).

### 3.4. Inflammatory Parameters

The levels of the TGF-β_1_ in feces of infants with NIM-CMPA were quite similar compared with values obtained in the control group, and statistical differences between both groups were not found (Table 2). In one infant of the control group and in three of the NIM-CMPA group, the levels of TGF-β_1_ were below the lower limit of quantification (0.62 pg/mL). A significant negative correlation between fecal levels of butyric acid and excretion of TGF-β_1_ was observed (*r* = 0.53, *p* = 0.04), but only in the group of infants with NIM-CMPA (Figure S3).

Fecal calprotectin concentrations in allergic infants did not show statistical differences with those found in control infants (Table 2). Calprotectin levels in the samples from the three infants that did not acquire tolerance, after the elimination diet, were no different from the levels found in the remaining NIM-CMPA patients, nor in the infants in the control group.

## 4. Discussion

NIM-CMPA is difficult to diagnose because clinical symptoms appear with a delayed onset and no specific diagnostic tests are available [20]. For this reason, there are only a few studies focusing on non-IgE mediated cases [10], and almost none of them consider the description of gut microbiota. The development of the intestinal microbiota and immune system could be playing a critical role in this condition, which affects children during their first two years of life. The important association between GI microbiota and food allergies in early infancy has been clearly pointed to in recent years [6,7,21], although the precise mechanisms of desensitization and interaction with the host remains poorly understood in most cases.

Our study was designed to evaluate intestinal microbiota and fecal associated parameters in infants with NIM-CMPA, under a milk elimination diet, compared to healthy infants, on an unrestricted diet, in an effort to establish potential links among microbiota and its metabolites, main feeding sources, and tolerance acquisition. The importance of formula selection for the management of infants with CMPA (both IgE and non-IgE mediated) and the acquisition of tolerance has been previously stated in studies by Berni Canani and colleagues, who demonstrated that an EHF supplemented with a probiotic (*Lactobacillus rhamnosus* GG) was able to accelerate tolerance acquisition in infants with CMPA [22,23]. However, the microbiota was not analyzed in these works. In our study, we observed that only those infants who were consuming rice hydrolyzed formulas did not develop clinical tolerance after six months of an exclusion diet, and presented significant differences in their microbiota with respect to those who outgrew their CMPA. To the best of our knowledge, this is the first study focusing exclusively on NIM-CMPA. A recent work has also been published in NIM-CMPA patients, but in that case, in suspected not challenge-proven cases [10]. In that interventional study, only a few groups of fecal microorganisms (bifidobacteria and *Clostridium coccoides* group (reclassified now as *Blautia coccoides*, belonging to the clostridial cluster XIVa)) were evaluated by fluorescence in situ hybridization. In our study, we obtain detailed insights into the microbiota composition using next-generation sequencing (NGS) techniques. We found that infants with NIM-CMPA that consumed vegetable protein-based formulas had less *Coriobacteriaceae*. Specifically, those fed with rice formulas presented low abundances of representatives of *Coriobacteriaceae* and *Bifidobacteriaceae* in their fecal samples. Members of *Bifidobacteriaceae* (in particular *Bifidobacterium*) and *Coriobacteriaceae* (mainly *Collinsella*) have been reported to be highly prevalent from the early years of life [21,24]. *Coriobacteriaceae*, and certainly the genus *Collinsella*, were present in higher rates in infants with NIM-CMPA that consumed an EHF in our study. Although the biology of these bacteria is still largely ignored [21], experiments in vitro in human models showed that both *Collinsella* spp. and *Bifidobacterium* spp. are the major lactose utilizers in the human gut [24]. In fact, lactose is present is some of the commercial EHFs, but not in vegetable ones. In our work, a positive correlation was found between *Coriobacteriaceae* and butyrate levels. This may be explained by the stimulation of certain butyrate producers, such as members of the *C. coccoides* group, through cross-feeding mechanisms [25] that are triggered by end fermentation metabolites, which are produced by *Coriobacteriaceae*.

We also observed statistically higher levels of BCFAs (isobutyric and isovaleric acids) in infants with NIM-CMPA as compared with infants in the control group. These minor SCFAs have often been associated with protein breakdown [26]. Previous studies of IgE-mediated CMPA revealed fecal concentrations and percentages of butyric acid and BCFAs higher in infants with CMPA than in healthy infants [27].

The most outstanding difference between allergic and healthy infants, in terms of fecal microbiota composition, in our study was the reduction in the relative proportions of the phylum Bacteroidetes, especially the family *Bacteroideaceae* and the genus *Bacteroides*, in the infants with NIM-CMPA (both tolerant and non-tolerant) after the restricted diet period. Bacteroidetes perform metabolic conversions that are essential for the host, often related to the degradation of proteins [24]. Some species may play an important role in protein metabolism since they have proteolytic activity. The association of *Bacteroides* with a high consumption of fat and proteins of animal origin has been previously mentioned [28,29]. It has been described that in the mature intestine of the child, by two to three years of age, the microbiota composition mainly consists of *Bacteroideaceae*, as well as *Lachnospiraceae* and *Ruminococcaceae* members [30]. Other conditions during infancy, such as antibiotic exposure, preterm birth, or cesarean section delivery, have been related with reduced abundance of Bacteroidetes [31,32,33]. Furthermore, in adults, this group of microorganisms has been reported to be affected in some digestive pathologies, such as irritable bowel syndrome [34].

The precise mechanisms leading to tolerance in NIM-CMPA cases are not yet elucidated. Several immunological mechanisms may be responsible for the non-IgE reactions. Th1/Th2 imbalances are assumed to have an impact, but both humoral and/or cell-mediated mechanisms may be implicated and induce symptoms [35]. The regulatory cytokine TGF-β_1_ is known to induce T-cell suppression, contributing to the downregulation of inflammatory processes. The expression of this growth factor in the intestinal mucosa increases with age during infancy [35]. In the present study, significant differences in the fecal levels of TGF-β_1_ between infants diagnosed with NIM-CMPA and healthy infants were not found. Additionally, the three infants with persistent CMPA after the second SOC showed values in the same range as those that acquired tolerance. In a previous study of patients with NIM-CMPA, a higher frequency of circulating regulatory T (Treg) cells were found in children who, after a milk-free period, outgrew their allergy compared to those who maintained clinically active hypersensitivity [36]. The authors suggested that the suppressive action of cow’s milk-specific Treg cells was exerted partly by direct cell–cell contact, and partly by production of TGF-β. In our study, it should be taken into consideration that fecal samples were taken after a period of six months of a dairy-free diet, during which most of the participants became tolerant, indicating that the underlying inflammatory condition of the gut may have normalized. Contrary to what we expected, we found a moderate, but significant, negative correlation between butyric acid and TGF-β_1_ fecal levels in the group of infants with NIM-CMPA. Although we do not have a clear explanation at this stage, we postulate that the relationship between butyric acid levels and TGF-β_1_ might be related to the role of the TGF-β receptor. Although this needs to be explored further in future studies, the activity of the type 1 receptor for TGF-β, rather than the cytokine itself, has already been suggested as being related with the pathogenesis of NIM-CMPA [37]. Furusawa and colleagues demonstrated, for the first time in 2013, that butyrate induce differentiation of colonic Treg cells, which have a central role in the suppression of inflammatory and allergic responses [38]. Butyrate exerts anti-inflammatory effects, through epigenetic mechanisms, influencing immune system development and function. The positive role of butyrate epigenetic effects on children’s health has been previously highlighted [39].

Calprotectin levels vary with age, and therefore different studies on children have used diverse cut-off values [40]. As observed previously by others [41], differences in calprotectin concentrations between healthy and allergic infants after a CMP elimination diet were not found in our study, probably due to most of the patients outgrowing their allergy after the therapeutic diet restriction stabilization period.

Patterns of formula selection in infants with CMPA are changing with an increased use of soy and rice hydrolyzed formulas [23,42]. Actually, more information is needed for the choice of formula substitutes for children with CMPA [5]. Our findings support the recommendations of the GI Committee of ESPGHAN [3], and show that the use of a vegetable dietary regimen may not be conducive to achieving oral tolerance, due to the absence of exposure to immunomodulatory peptides and the shaping of the gut microbial communities. We realize the limitations of the study, namely the small number of patients included in each group. Therefore, the data found in this pilot study need to be confirmed in a larger population, especially since some studies in the past did not find differences in tolerance acquisition rates between the choice of different vegetable (rice, soy, and protein) formulas with respect to EHFs [43,44].

Our results indicate that the type of formula consumed in early life can determine the composition and diversity of the microbiota established. These preliminary data on NIM-CMPA still have to be taken with caution due to the high inter-individual variability in the microbiota among infants in this age period [21]. Although, with the present data, we hypothesize that differences in a child’s main diet may influence the intestinal microbiota and its metabolic products, and, ultimately, influence tolerance acquisition, which has an important impact on clinical practice in NIM-CMPA.

## Figures and Tables

**Table 1:** Significant differences (at different taxonomic ranks) in fecal microbial abundances (%) between tolerant and non-tolerant infants with non-IgE mediated cow’s milk protein allergy (NIM-CMPA) after a period with a diet free of cow’s milk protein (CMP).

Phylum	*p* Value ^a^	Relative Abundance ^b^	
		Non-Tolerant CMPA Infants (*n* = 3)	Tolerant CMPA Infants (*n* = 14)
Actinobacteria	0.002	0.428 ± 0.200	21.775 ± 15.731
**Family**			
*Bifidobacteriaceae*	0.002	0.087 ± 0.141	17.705 ± 15.513
*Coriobacteriaceae*	0.009	0.266 ± 0.224	3.990 ± 4.087
**Genus**			
*Bifidobacterium*	0.002	0.087 ± 0.141	17.680 ± 15.506

^a^ Significance was considered below a *p* value of 0.05, multiple hypothesis test correction of Benjamini and Hochberg was applied with a false discovery rate (FDR) of 0.25. ^b^ Mean relative abundance ± standard deviation.

**Table 2:** Levels of main short-chain fatty acids (SCFAs), branched chain fatty acids (BCFAs), transforming growth factor-β_1_ (TGF-β_1_), and calprotectin excreted in feces of infants with non-IgE mediated cow’s milk protein allergy (NIM-CMPA) and infants in the control group.

Median (IQR) ^a^	Infants		*p* Value ^b^
	NIM-CMPA (*n* = 17)	Control (*n* = 10)	
BCFAs (µmol/g)	5.13 (3.08–6.52)	2.59 (1.94–3.37)	0.03
Acetic (µmol/g)	54.88 (48.05–89.63)	68.61 (50.49–69.96)	0.90
Propionic (µmol/g)	16.19 (13.09–21.48)	15.64 (11.66–24.06)	0.94
Butyric (µmol/g)	17.59 (12.74–21.41)	12.88 (6.14–14.3)	0.06
TGF-β_1_ (pg/mL)	1774.79 (1153.10–3810.88)	1496.29 (382.23–5820.20)	0.73
Calprotectin (µg/g)	47.25 (28.80–106.10)	68.40 (30.38–76.73)	1.00

^a^ Concentrations represent median and interquartile ranges (IQR). ^b^ Mann–Whitney U test. Significance was set at *p* = 0.05.

## Supplementary Materials

The following are available online at http://www.mdpi.com/2072-6643/10/10/1481/s1, Figure S1: Microbial composition at the phylum level in fecal samples of infants with non-IgE mediated (NIM) cow’s milk protein allergy (CMPA) and non-allergic control infants. Figure S2: Regression of butyric acid levels on *Coriobacteriaceaea* abundances in all infants of the study (allergic and control; *n* = 27). Figure S3: Correlation between transforming growth factor (TGF)-β_1_ and butyric acid levels in feces of infants with non-IgE mediated (NIM) cow’s milk protein allergy (CMPA) (*n* = 17).
