# Supplementary material for: Microbiota and Derived Parameters in Fecal Samples of Infants with Non-IgE Cow’s Milk Protein Allergy under a Restricted Diet
Source: Nutrients. 2018 Oct 11;10(10):1481. doi: 10.3390/nu10101481 (PMC6213916; doi:10.3390/nu10101481)
Supplement: Supplementary file 1 [file nutrients-10-01481-s001.pdf]

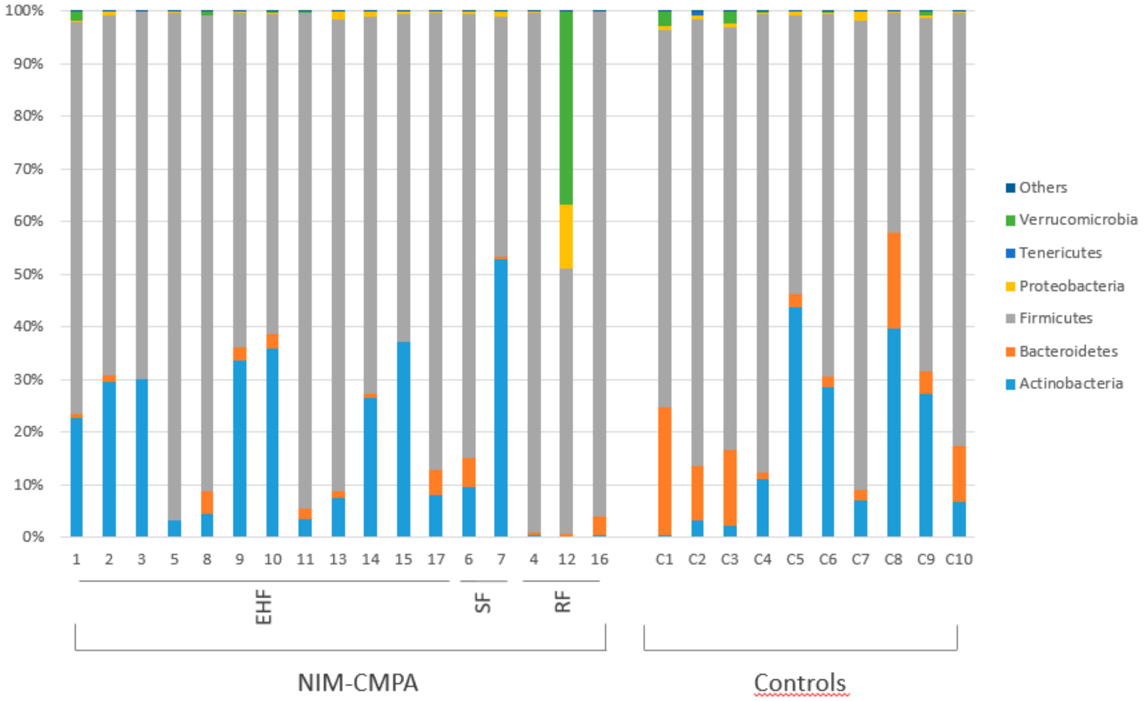

Figure S1. Microbial composition at the phylum level in fecal samples of non-IgE mediated (NIM) cow´s milk protein allergy (CMPA) infants and non-allergic control infants. Relative abundances are represented as percentages (%). Only those phyla with relative abundances ≥ 0.5% are showed. Type of infant feeding (EHF, extensively hydrolyzed formula; SF, soy formula; RF, rice formula) is underlined.

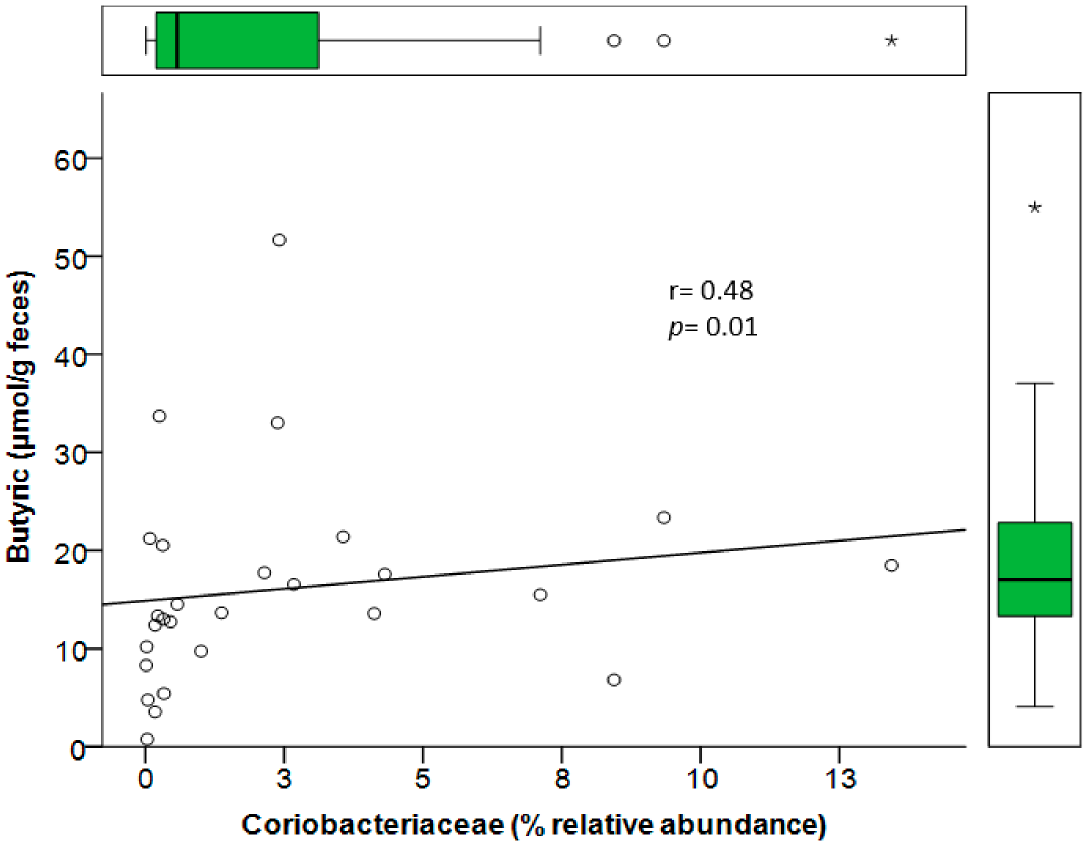

Figure S2. Regression of butyric acid levels on *Coriobacteriaceaea* abundances in all infants of the study (allergic and controls; n = 27). Spearman's rank correlation coefficient r and two-tailed p value are indicated. Representations of the two variables positive associated are also shown by central green rectangles that reflect the interquartile ranges (IQR). The lines inside the rectangles indicate the medians and the whiskers the maximum and minimum values. The dots outside the rectangles are suspected outliers (> 1.5 × IQR) meanwhile the asterisk designates an extreme value (> 3 × IQR).

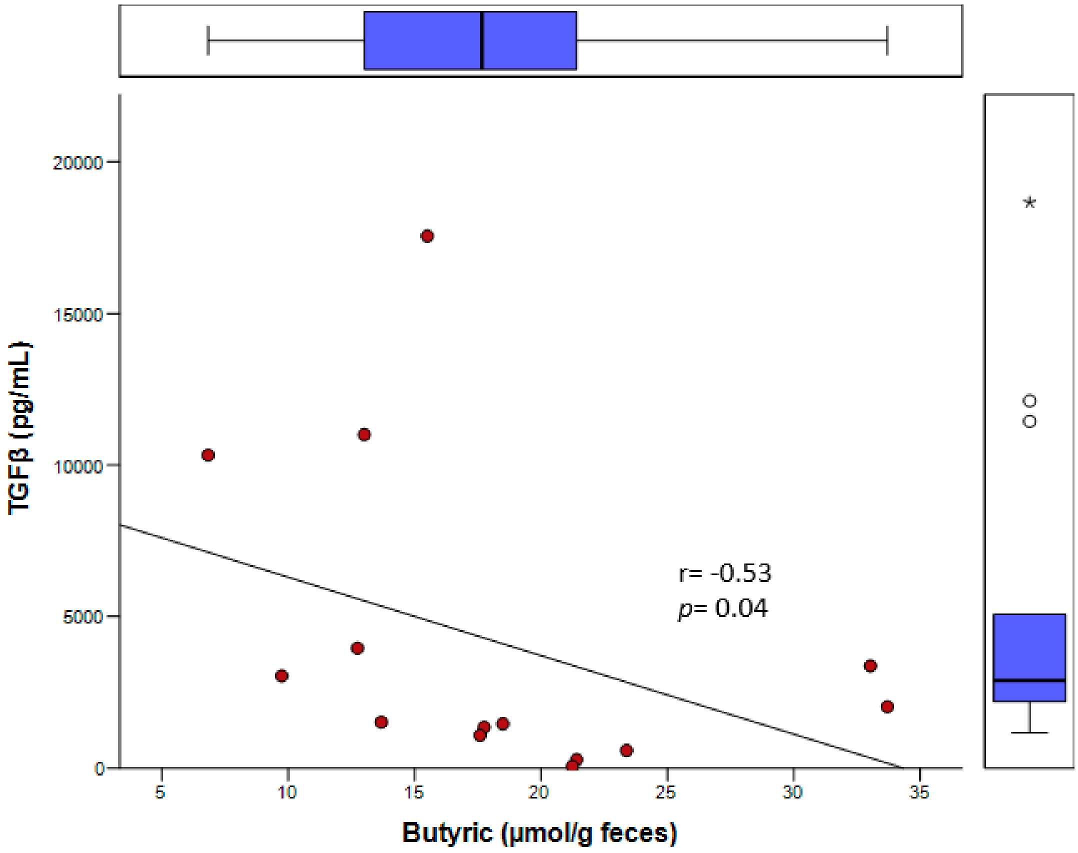

Figure S3. Correlation between transforming growth factor (TGF)-β_1 and butyric acid levels in feces of infants with non-IgE mediated (NIM) cow´s milk protein allergy (CMPA) (n = 17). Correlation is significant at the 0.05 level (2-tailed). Spearman's rank correlation coefficient r and p value are both indicated. Representations of the two variables negatively associated are also shown by central violet rectangles that reflect the interquartile ranges (IQR). The lines inside the rectangles indicate the medians and the whiskers the maximum and minimum values. The dots outside the rectangles are suspected outliers (> 1.5 × IQR) meanwhile the asterisk designates an extreme value (> 3 × IQR).
